# Moving Backgrounds Massively Change the Apparent Size, Shape and Orientation of Flashed Test Squares

**DOI:** 10.1177/2041669517737561

**Published:** 2017-11-16

**Authors:** Stuart Anstis, Patrick Cavanagh

**Affiliations:** University of California, San Diego, La Jolla, CA, USA; Dartmouth College, Hanover, NH, USA

**Keywords:** illusion, motion, shape, spatial vision

## Abstract

A random-dot background was expanded and contracted, and rotated, or expanded in one dimension while contracting on the other, or skewed back and forth horizontally. Squares that were flashed at the reversal points of these affine pattern distortions, aligned to edges in the texture, showed massive changes in size and shape.

## Introduction

Various misperceptions of position arise when one object is briefly flashed on or near a moving object or texture (for a review, see Eagleman & Sejnowski, 2007). Interestingly, in all these motion-induced position shifts, the illusory displacement is equivalent to the distance the moving object or background would travel in 80 to 100 ms (e.g., Öğmen et al., 2004). This shift might be compensating for a delay of about 100 ms (Nijhawan, 1994), or it might be the average position of the moving object in the 100 ms after the test is presented (Eagleman & Sejnowski, 2007). We now show a new version from this family of motion-induced position shifts based on our earlier flash-grab effect (Cavanagh & Anstis, 2013). Shapes are perceptually sucked along in the direction of subsequent (not preceding) background motion. This new version alters the perceived size, shape and orientation of test squares much more than any previous similar illusions. The extra potency of these distortions challenges models of motion-induced position shifts because it breaks the 100 ms barrier – contours are shifted by much farther than the background travels in 100 ms.

**Figure 1.**
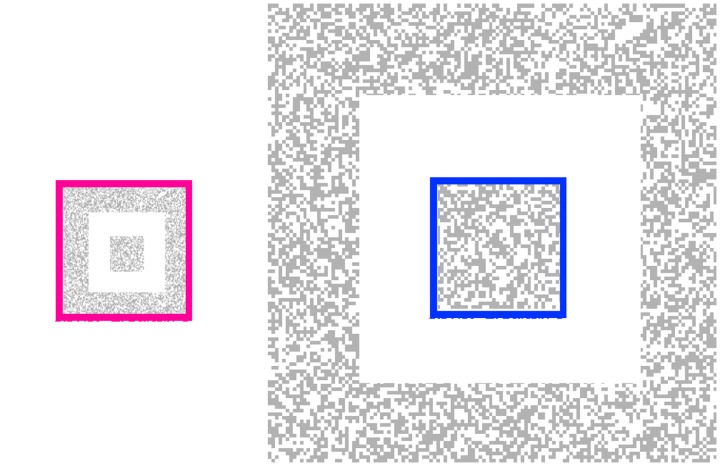
Two end frames from Movie 1a where the red square and blue square are presented. In the movie, the red square appears to be about twice the size of the blue square. In these static frames, however, there is little or no difference indicating that the perceived difference in the movie does not come from a simple static size contrast between the colored squares and the textured pattern.

Experiment 1 measured the illusory size change with a matching method. A square bull’s-eye of random dots with an inner square and an outer annulus was presented that contracted and expanded between 2° and 8° outer diameter, a fourfold size change, at a rate of 1 Hz. When the background was at its maximum size, a blue, outline square was flashed that aligned with the edge of the inner texture square (2° across). When the background was at its smallest size, a red outline square was flashed aligned to the outer edge of the outer texture annulus (now also 2° across). Although the red and blue squares were congruent, the red square appeared much larger than the blue square (online Movie 1(a)). Adjustable, concentric red and blue matching squares were flashed in alternation, 5° to the right of the display. Observers adjusted the sizes of the matching squares to be equal to those of the flashing test squares. The size ratio of the matching squares was 2.20 ± 0.53 (mean of 3 participants) with the red matching square set to more than twice the size of the blue matching square (see online Movie 1(c)). Note that the perceptual distortion induced into the flashed square is opposite to the instantaneous physical size of the background. Thus, when the background is largest, the flashed blue square appears minimized in size. Figure 1 shows that that any static size contrast had a minimal effect.

In online Movie 1(b), the background also rotates through 90°, which makes the test squares appear to twist around in the opposite direction. The twists are made more visible by adding a small marker in one corner. To measure the amount of induced rotation, participants adjusted the orientation of adjustable concentric matching squares. The average match was a 56.3° ± 4.7° rotation (mean of 3 participants) between the red and blue squares (see online Movie 1(d)).

We extended these shape change effects to skew distortion (online Movie 2(a)), and the effect is again remarkably large. The change in the matched apparent vertical in the skew case was 22.7° ± 2.2° (mean of 2 participants), between the leftward and rightward tilt.

These motion-induced distortions are substantially larger than previous reports and this large size constrains possible models of the effects. In order to compare the size of these effects to each other and to previous findings, we take the distance of the contour displacements that underlie the shape distortion and convert these into equivalent travel times of the background at the location of the borders that are shifted, as if the borders had moved along with the background during that time. Previous studies have used this same conversion (e.g., Eagleman & Sejnowski, 2007; Öğmen et al., 2004) to normalize for the effect of speed as position shifts scale linearly with speed up to a saturation value (e.g., Cavanagh & Anstis, 2013). For the size distortions here, the borders shift by the equivalent of 155 ± 44 ms of background travel. The rotation effect of 56° is also larger than largest previous report of motion-induced rotation, 45.7° (Cavanagh & Anstis, 2013), and equivalent to 156 ± 13 ms in terms of travel time. The shape distortion in the skew condition is equivalent to 142 ± 16 ms. On this normalized scale, our results for the three conditions here stand out as significantly larger than anything previously reported.

These time values also constrain models of the effect. Some authors have attributed these shifts to differential latencies of up to 80 ms between moving and flashed items (see Öğmen et al., 2004). Others suggest that the shifts represent a compensation for neural delays so that the perceived location of a moving stimulus matches its actual position with an extrapolation to make up for distance the motion travelled during these delays (e.g., Nijhawan, 1994). Eagleman and Sejnowski (2007) proposed that the shifts represent the average location of a moving object with the averaging beginning at the moment of the test flash. All of these proposals are challenged by an equivalent travel time of 150 ms as there is little evidence that either neural delays or time averaging windows exceed 100 ms.

Either these mechanisms need some revision to cope with these large time constants or possibly shape contrast illusions (Suzuki & Cavanagh, 1998), size contrast or induced motion may contribute.

## Author Note

Patrick Cavanagh is also affiliated with Glendon College, Toronto, ON, Canada.

## References

[bibr1-2041669517737561] Cavanagh P. Anstis S. (2013) The flash grab effect. Vision Research 91: 8–20. 23872166 10.1016/j.visres.2013.07.007 PMC5047291

[bibr2-2041669517737561] Eagleman D. M. Sejnowski T. J. (2007) Motion signals bias localization judgments: A unified explanation for the flash-lag, flash-drag, flash-jump, and Frohlich illusions. Journal of Vision 7: 3. 10.1167/7.4.3 PMC2276694 17461687

[bibr3-2041669517737561] Nijhawan R. (1994) Motion extrapolation in catching. Nature 370: 256–257. 10.1038/370256b0 8035873

[bibr4-2041669517737561] Öğmen H. Patel S. S. Bedell H. E. Camuz K. (2004) Differential latencies and the dynamics of the position computation process for moving targets. Vision Research 44: 2109–2128. 15183678 10.1016/j.visres.2004.04.003

[bibr5-2041669517737561] Suzuki S. Cavanagh P. (1998) A shape-contrast effect for briefly presented stimuli. Journal of Experimental Psychology: Human Perception and Performance 24: 1315–1341. 9778826 10.1037//0096-1523.24.5.1315

